# MAPK6-AKT signaling promotes tumor growth and resistance to mTOR kinase blockade

**DOI:** 10.1126/sciadv.abi6439

**Published:** 2021-11-12

**Authors:** Qinbo Cai, Wolong Zhou, Wei Wang, Bingning Dong, Dong Han, Tao Shen, Chad J. Creighton, David D. Moore, Feng Yang

**Affiliations:** 1 Department of Molecular and Cellular Biology, Baylor College of Medicine, Houston, TX 77030, USA.; 2 Center of Gastrointestinal Surgery, the First Affiliated Hospital of Sun Yat-sen University, Guangzhou 510080, China.; 3 Department of Thoracic Surgery, Xiangya Hospital of Central South University, Changsha 410008, China.; 4 Department of Medicine, Baylor College of Medicine, Houston, TX 77070, USA.; 5 Dan L. Duncan Comprehensive Cancer Center, Baylor College of Medicine, Houston, TX 77030, USA.; 6 Nutritional Sciences and Toxicology, University of California, Berkeley, Berkeley, CA 94720, USA.

## Abstract

Mitogen-activated protein kinase 6 (MAPK6) is an atypical MAPK. Its function in regulating cancer growth remains elusive. Here, we reported that MAPK6 directly activated AKT and induced oncogenic outcomes. MAPK6 interacted with AKT through its C34 region and the C-terminal tail and phosphorylated AKT at S473 independent of mTORC2, the major S473 kinase. mTOR kinase inhibitors have not made notable progress in the clinic. Our identified MAPK6-AKT axis may provide a major resistance pathway. Besides repressing growth, inhibiting MAPK6 sensitized cancer cells to mTOR kinase inhibitors. MAPK6 overexpression is associated with decreased overall survival and the survival of patients with lung adenocarcinoma, mesothelioma, uveal melanoma, and breast cancer. MAPK6 expression also correlated with AKT phosphorylation at S473 in human cancer tissues. We conclude that MAPK6 can promote cancer by activating AKT independent of mTORC2 and targeting MAPK6, either alone or in combination with mTOR blockade, may be effective in cancers.

## Teaser

MAPK6-AKT signaling promotes tumor growth and resistance to mTOR kinase inhibitors.

## INTRODUCTION

Mitogen-activated protein kinase 6 (MAPK6) is considered an atypical MAPK because its activation loop contains a Ser-Glu-Gly (SEG) motif compared to the conserved Thr-X-Tyr (TXY) motif in the canonical MAP kinases (*1*). In the canonical pathway, MAPK activation is dependent on the MAPK kinase kinase (MAPKKK or MAP3K) and MAPK kinase (MAPKK or MAP2K) signaling cascade (*2*, *3*). In contrast, MAPK6 appears to be phosphorylated at S189 of the SEG motif and activated by the group I p21-activated protein kinases (PAK1/2/3) (*4*, *5*). MAPK6 can phosphorylate several protein substrates, including MAP kinase–activated protein kinase 5 (MAPKAPK5 or MK5) at T182 (*6*, *7*), steroid receptor coactivator 3 (SRC-3) at S857 (*8*), and tyrosyl DNA phosphodiesterase 2 (TDP2) at S60 (*9*).

While the roles of canonical MAPKs, such as MAPK3/1 [extracellular signal–regulated kinase 1/2 (ERK1/2)], MAPK11-14 (p38β/γ/δ/α), and MAPK8-10 (c-Jun N-terminal kinase 1/2/3), are well established in carcinogenesis and tumor progression (*2*, *3*), the role of MAPK6 in human cancers remains unclear. The best-described tumor-promoting activity of MAPK6 is enhancing the migration/invasion/metastasis of cancer cells, including those of lung, head and neck, breast, and cervix, as well as the human umbilical vein endothelial cells (HUVECs) and vascular smooth muscle cells (VSMCs) (*8*, *10*–*15*). MAPK6 also promoted HUVEC, VSMC, and cervical cancer cell growth (*10*, *13*, *14*). In contrast, MAPK6 was reported to repress fibroblast cell cycle progression (*16*), melanoma and intrahepatic cholangiocarcinoma cell proliferation, and melanoma cell migration (*17*, *18*), all of which support a tumor suppressor function. Thus, MAPK6 function in human cancers, especially its role in regulating cancer growth, remains elusive.

In this study, we report that MAPK6 overexpression induces oncogenic outcomes, including transforming “normal” epithelial cells into anchorage-independent growth and enhancing cancer cell growth in an AKT-dependent manner. MAPK6 binds and phosphorylates AKT at S473 independent of the major S473 kinase of AKT, mechanistic target of rapamycin (mTOR) complex 2. Accordingly, besides inhibiting growth, repressing MAPK6 also greatly sensitizes cancer cells to mTOR kinase blockade. This MAPK6-AKT pathway may provide a major route for cancer resistance to mTOR kinase inhibition, which may contribute to the lack of notable progress of using mTOR kinase inhibitors in the clinic. We conclude that MAPK6 can promote cancer by activating AKT and that targeting MAPK6, either alone or in combination with mTOR kinase blockade, may provide effective therapeutic approaches for cancer.

## RESULTS

### MAPK6 overexpression transforms normal epithelial cells into anchorage-independent growth

**Fig. 1.**
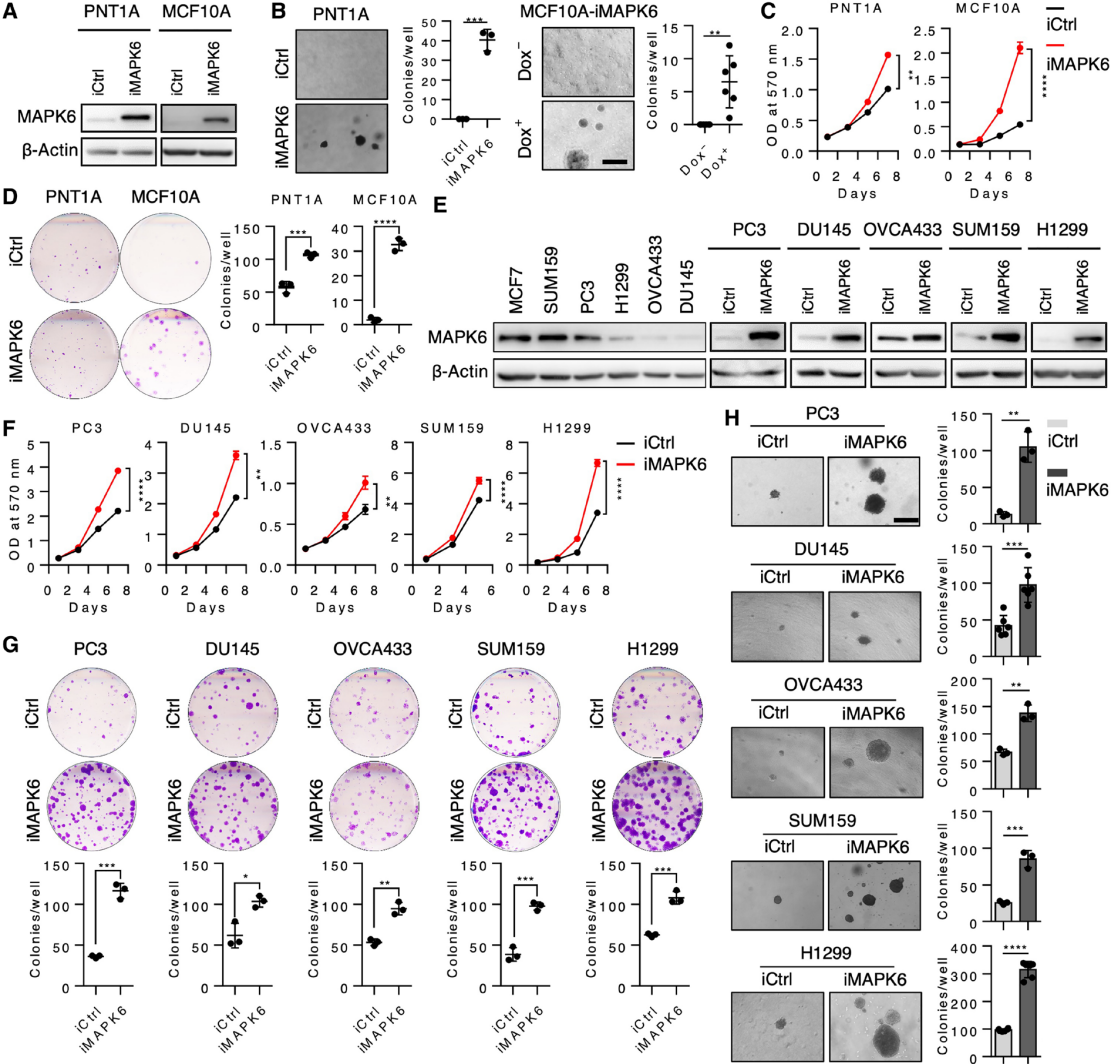
MAPK6 overexpression promotes tumor cell growth. (**A**) Western blots on Dox-induced (48 hours) overexpression of MAPK6 (iMAPK6) or control (iCtrl) in the engineered PNT1A and MCF10A cells. (**B**) Soft-agar assays on PNT1A-iMAPK6 and iCtrl cells induced with Dox (1 μg/ml) (left) and the MCF10A-iMAPK6 cells treated with (+) or without (−) Dox (right). Scale bar, 500 μm. (**C**) Proliferation assays on the engineered PNT1A and MCF10A cells with Dox-induced overexpression of MAPK6 (iMAPK6) or control (iCtrl). Quantification data as means ± SD, *P* values by two-way ANOVA. (**D**) Plate colony formation assays on cells in (C). (**E**) Western blots on endogenous and Dox-induced (48 hours) overexpression of MAPK6 in different cancer cell lines. (**F**) Proliferation assays on engineered cells with Dox-induced overexpression of MAPK6 (iMAPK6) or control (iCtrl). Quantification data as means ± SD, *P* values by two-way ANOVA. (**G**) Plate colony formation and (**H**) Soft-agar assays on cell lines described in (F). Scale bar, 500 μm. Dox (1 μg/ml) was used to induce MAPK6 in all the above experiments. Quantification data as means ± SD, *P* values by Student’s *t* test except as otherwise indicated. *P ≤ 0.05, **P ≤ 0.01, ***P ≤ 0.001, ****P ≤ 0.0001. Data are representative of at least three independent experiments.

Previous studies have reported both tumor-promoting and tumor-inhibiting activities of MAPK6. To assess MAPK6 function, we first overexpressed it in the immortalized normal human prostate epithelial PNT1A cells and breast epithelial MCF10A cells in a doxycycline (Dox)–inducible manner (Fig. 1A). We then assessed MAPK6 ability to transform these cells into anchorage-independent growth in the soft-agar assays. Similar to the robust activities of the closely related MAPK4 in transforming PNT1A cells (*19*), MAPK6 overexpression transformed both PNT1A cells and MCF10A cells into anchorage-independent growth (Fig. 1B). In accord with this identified oncogenic activity of MAPK6, MAPK6 overexpression also promoted the growth, including the clonogenic growth of these normal PNT1A and MCF10A cells (Fig. 1, C and D).

### MAPK6 promotes tumor cell growth

MAPK6 has been reported to exhibit tumor-promoting activities by enhancing tumor cell migration/invasion but not growth (*8*). In sharp contrast, our data (Fig. 1, A to D) support a growth-promoting role of MAPK6 in cancer cells. To further assess MAPK6 function in human cancer cells, we next examined the impact of Dox-inducible MAPK6 overexpression in several diverse human cancer cell lines with low to high endogenous levels of MAPK6, including the prostate cancer PC3 and DU145, ovarian cancer OVCA433, breast cancer SUM159, and non–small cell lung cancer H1299 cells (Fig. 1E). Dox-induced MAPK6 expression significantly promoted the growth, including the clonogenic growth and the anchorage-independent growth, of all these tumor cells (Fig. 1, F to H).

**Fig. 2.**
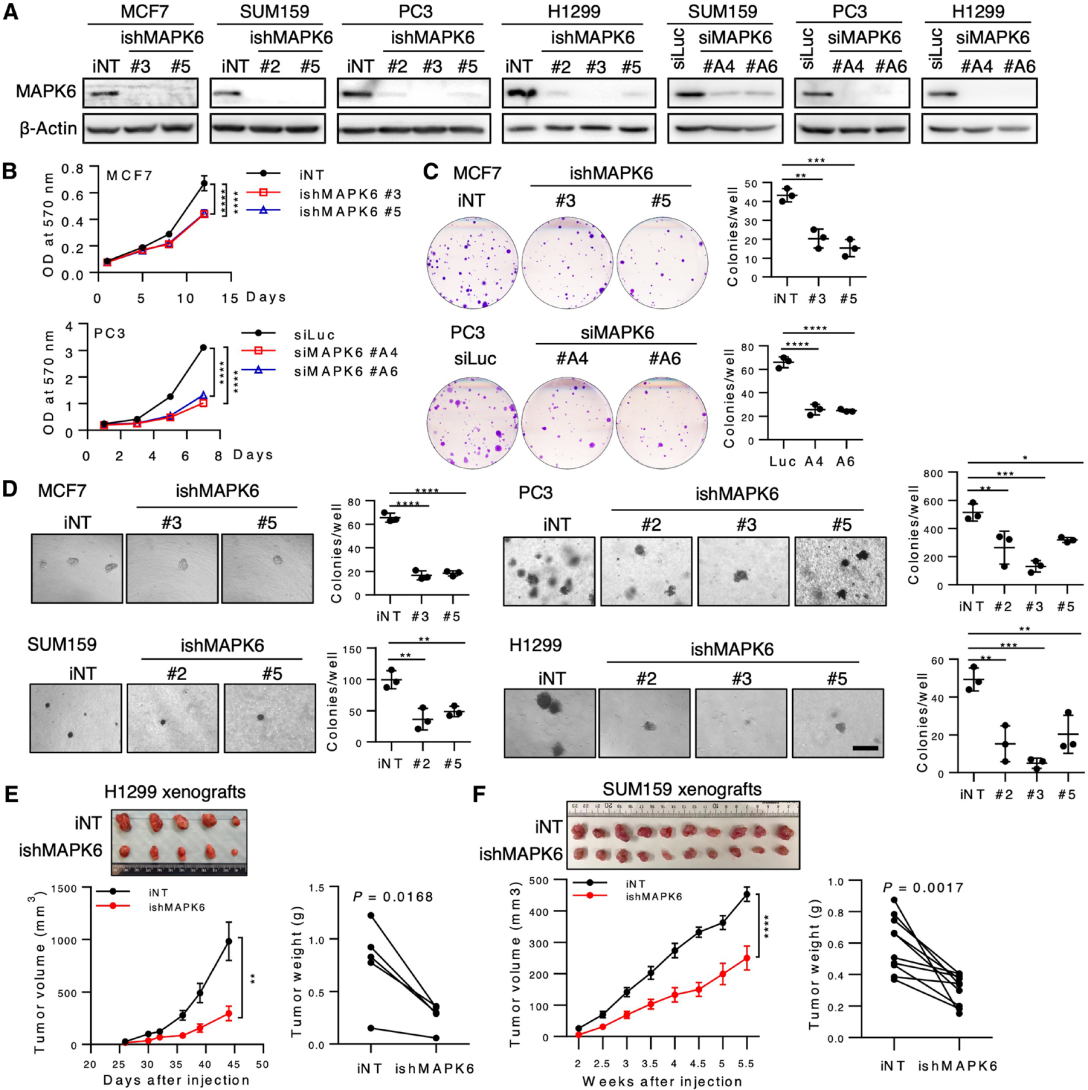
Knockdown of MAPK6 inhibits tumor growth. (**A**) Western blots on Dox-induced knockdown of MAPK6 by shRNA (ishMAPK6, #2, #3, or #5) or transient knockdown of MAPK6 by siRNA (siMAPK6, #A4, #A6) in cancer cell lines. iNT, inducible nontargeting control; siLuc, siRNA against luciferase. (**B**) Proliferation assays on MCF7 cells with Dox-induced knockdown (4 μg/ml) of MAPK6 (ishMAPK6) and PC3 cells 48 hours after being transfected with siRNAs against MAPK6 (siMAPK6). Quantification data as means ± SD, *P* values by two-way ANOVA. (**C**) Plate colony formation and (**D**) Soft-agar assays on cells as described in (A) and (B). Data as means ± SD, *P* values by one-way ANOVA with multiple comparisons corrected with Dunnett’s test. Scale bar, 500 μm. (**E**) H1299 (1 × 10^6^) or (**F**) SUM159 (2 × 10^6^) iNT or ishMAPK6 cells were injected into the left (iNT) and right (ishMAPK6) lateral flanks (H1299) or mammary fat pad (SUM159) of SCID mice, which also received Dox (4 mg/ml) in drinking water. Tumors were harvested as indicated. Shown are the tumors’ images, growth curve (means ± SEM, two-way ANOVA), and weights (paired Student’s *t* test). *P ≤ 0.05, **P ≤ 0.01, ***P ≤ 0.001, ****P ≤ 0.0001. Each xenograft pair represents an iNT versus an ishMAPK6 xenograft within the same mouse. Data are representative of at least two to three independent experiments. Photo credit: Qinbo Cai (E) and Dong Han (F), Baylor College of Medicine.

To further investigate the biological function of endogenous MAPK6, we performed small interfering RNA (siRNA) and/or Dox-induced short hairpin RNA (shRNA)-mediated knockdown of MAPK6 in the MAPK6-high or MAPK6-medium MCF7, SUM159, PC3, and H1299 cells (Fig. 2A). In agreement with the gain-of-function studies, shRNA or siRNA-induced knockdown of MAPK6 significantly inhibited MCF7, PC3, H1299, and SUM159 cell growth in the proliferation and clonogenic assays (Fig. 2, B and C, and fig. S1, A and B). Dox-induced shRNA knockdown of MAPK6 also greatly inhibited the anchorage-independent growth of all these cells in vitro and the H1299 and SUM159 xenograft growth in vivo (Fig. 2, D to F). Accordingly, Dox-induced ectopic expression of MAPK6 promoted PC3 xenograft tumor growth (fig. S1C). Together, our data support a tumor growth–promoting function of MAPK6.

### MAPK6 activates AKT

**Fig. 3.**
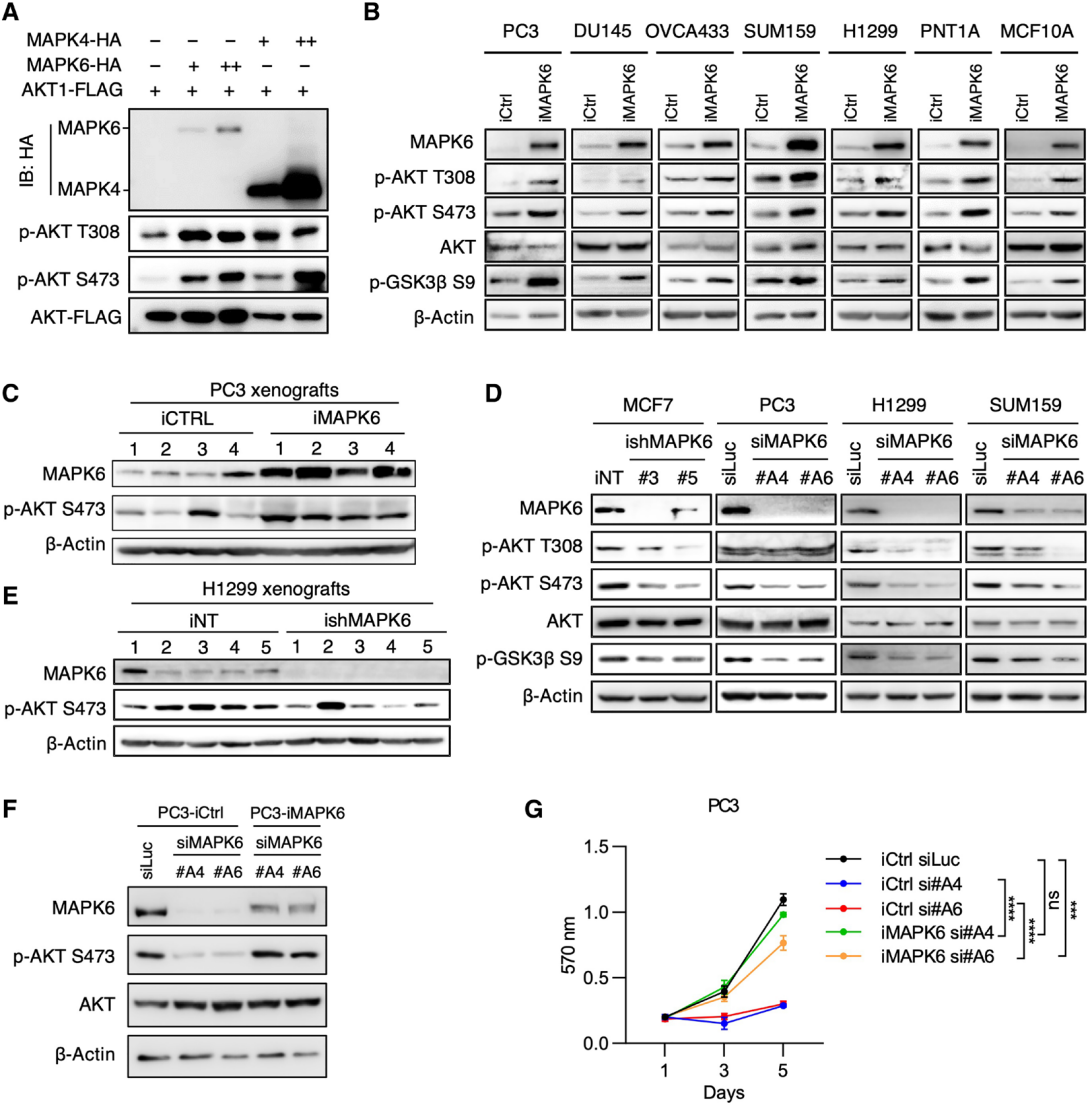
MAPK6 activates AKT. Western blots on (**A**) HEK293T cells 48 hours after the indicated transfections, (**B**) Dox-induced (1 μg/ml) (3 to 5 days) engineered PC3, DU145, OVCA433, SUM159, H1299, PNT1A, and MCF10A cells with overexpression of MAPK6 (iMAPK6) or control (iCtrl), (**C**) PC3 xenograft tumors as described in fig. S1C, and (**D**) MAPK6-knockdown MCF7, PC3, H1299, and SUM159 cells. The engineered MCF7 cells were treated with Dox (4 μg/ml) for 5 days for knockdown of MAPK6 (ishMAPK6 #3, #5) or control (iNT). The PC3, H1299, and SUM159 cells were transfected with siRNA against MAPK6 (siMAPK6 #A4, #A6) or luciferase control (siLuc). Seventy-two hours later, the cell lysates were prepared and applied in the Western blots. Western blots on (**E**) H1299 xenograft tumors as described in Fig. 2E, and (**F**) MAPK6-knockdown PC3 cells rescued with ectopic expression of MAPK6 versus control. The engineered PC3 cells with Dox-induced expression of MAPK6 (iMAPK6) and control (iCtrl) were transfected with siRNA against MAPK6 (siMAPK6 #A4, #A6) or luciferase control (siLuc). Seventy-two hours later, the cell lysates were prepared and applied in the Western blots. (**G**) Proliferation assays on cells as described in (F). Quantification data as means ±SD, *P* values by two-way ANOVA. ***P ≤ 0.001, ****P ≤ 0.0001. ns, not significant. Data are representative of at least two to three independent experiments.

MAPK6 is closely related to MAPK4 (*1*). Since MAPK4 can directly activate AKT to promote tumor growth (*19*, *20*), we examined whether MAPK6 also activates AKT. To directly compare MAPK4 and MAPK6 activities in regulating AKT phosphorylation, we cotransfected FLAG-tagged AKT together with increasing doses of hemagglutinin peptide (HA)–tagged MAPK6 or HA-tagged MAPK4 into the human embryonic kidney (HEK) 293T cells. The level of the ectopically overexpressed MAPK6 was substantially lower than that of MAPK4 (both detected by anti-HA Western blots; Fig. 3A), which agrees with a previous report on the rapid turnover of MAPK6 protein (*21*). Although expressed at lower levels, MAPK6 exhibited similar activities as MAPK4 in enhancing AKT phosphorylation at both T308 and S473 (Fig. 3A). Consistently, Dox-induced MAPK6 expression enhanced AKT phosphorylation/activation in PC3, DU145, OVCA433, SUM159, H1299, PNT1A, and MCF10A cells in vitro and PC3 xenografts in vivo (Fig. 3, B and C). In addition, knockdown of MAPK6 inhibited AKT phosphorylation/activation in MCF7, PC3, H1299, and SUM159 cells in vitro and H1299 xenografts in vivo (Fig. 3, D and E). Last, ectopic reexpression of MAPK6 into the MAPK6-knockdown PC3 cells largely rescued AKT phosphorylation and cell growth (Fig. 3, F and G). Together, these data support that MAPK6 phosphorylates/activates AKT.

### AKT activation is essential for the oncogenic and tumor-promoting activity of MAPK6

**Fig. 4.**
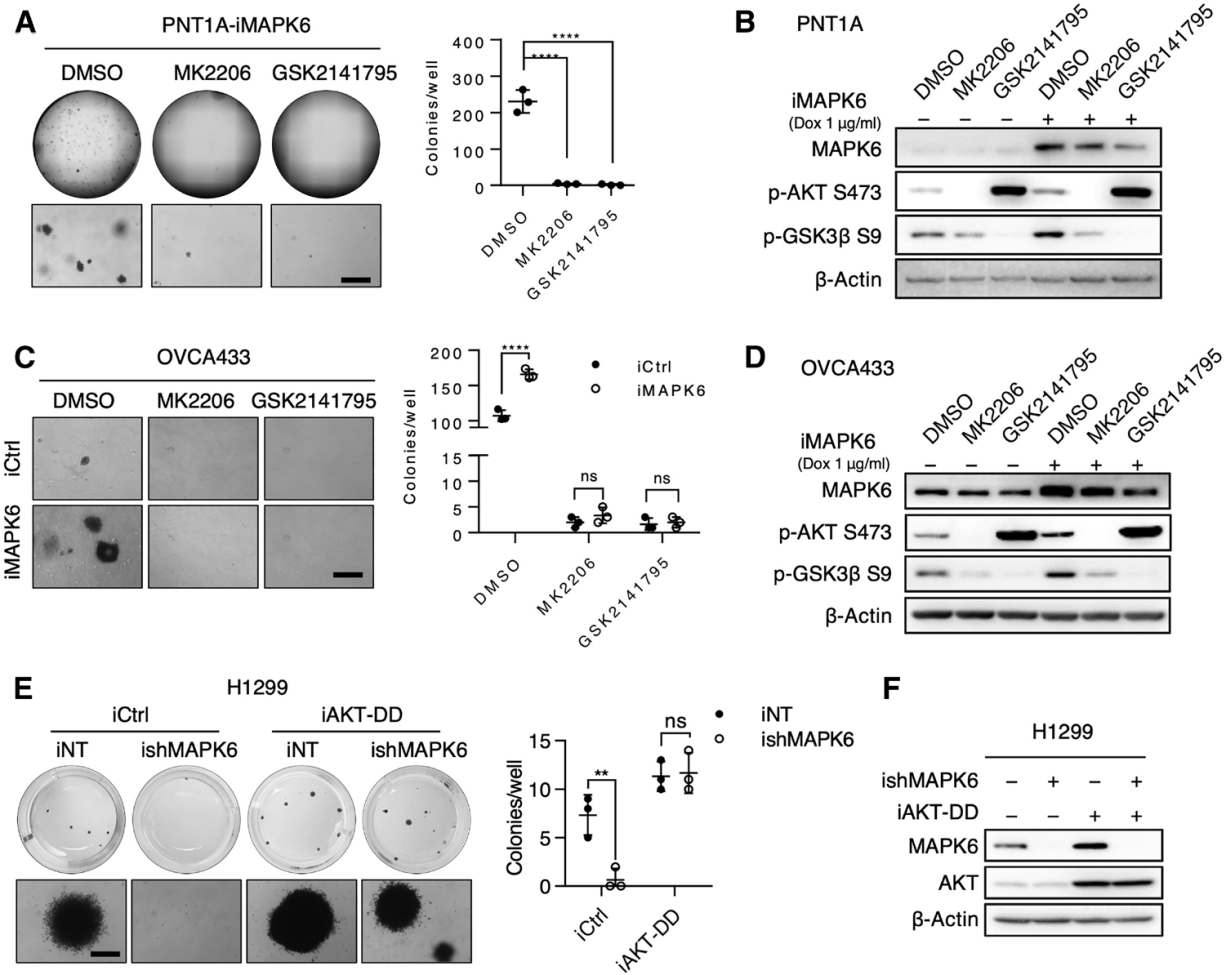
AKT activation is essential for the oncogenic and tumor-promoting activity of MAPK6. (**A**) Representative images (whole well and enlarged field) of soft-agar assays on Dox-induced (0.5 μg/ml) PNT1A-iMAPK6 cells treated with 2 μM of MK2206, GSK2141795, or dimethyl sulfoxide (DMSO). Quantification data as means ± SD, one-way ANOVA with multiple comparisons corrected with Dunnett’s test. (**B**) Western blots on PNT1A-iMAPK6 cells induced with Dox (+) or control (−) for 48 hours followed by treatments of 2 μM MK2206, 2 μM GSK2141795, or DMSO for 18 hours. (**C** and **D**) Similar studies as (A) and (B) were performed on the engineered OVCA433 cells. Data as means ± SD, two-way ANOVA with multiple comparisons corrected with Sidak’s test. (**E**) Representative images (whole well and enlarged field) of soft-agar assays on Dox-induced (1 μg/ml) engineered H1299 cells with overexpression of AKT1^T308D/S473D^ (iAKT-DD) versus control (iCtrl) with knockdown of MAPK6 (ishMAPK6) versus control (iNT). Quantification data as means ± SD, two-way ANOVA analysis as described in (C). (**F**) Western blots on the engineered H1299 cells in (E). The cells were treated with Dox (1 μg/ml) for 48 hours before Western blots analysis. **P ≤ 0.01. ****P ≤ 0.0001. ns, not significant. Data are representative of at least three independent experiments.

To assess the functional significance of AKT activation in mediating the oncogenic and growth-promoting activity of MAPK6, we performed soft-agar assays on Dox-induced PNT1A-iMAPK6 cells treated with AKT inhibitors MK2206, GSK2141795, or vehicle control. While MAPK6 overexpression transformed the PNT1A cells into anchorage-independent growth, MK2206 or GSK2141795 treatment largely abolished this growth (Fig. 4A). Western blots confirmed that both MK2206 and GSK2141795 repressed MAPK6-induced AKT activation as indicated by glycogen synthase kinase (GSK) 3β phosphorylation at S9 (Fig. 4B). Inhibiting AKT also blocked both basal and MAPK6-induced anchorage-independent growth of OVCA433 cells (Fig. 4, C and D). Last, overexpression of a constitutively active AKT1^T308D/S473D^ (AKT-DD) mutant rescued the anchorage-independent growth of MAPK6-knockdown H1299 cells (Fig. 4, E and F). Together, these data strongly support an essential role for AKT activation in mediating the oncogenic and tumor-promoting activities of MAPK6.

### MAPK6 phosphorylates AKT at S473

**Fig. 5.**
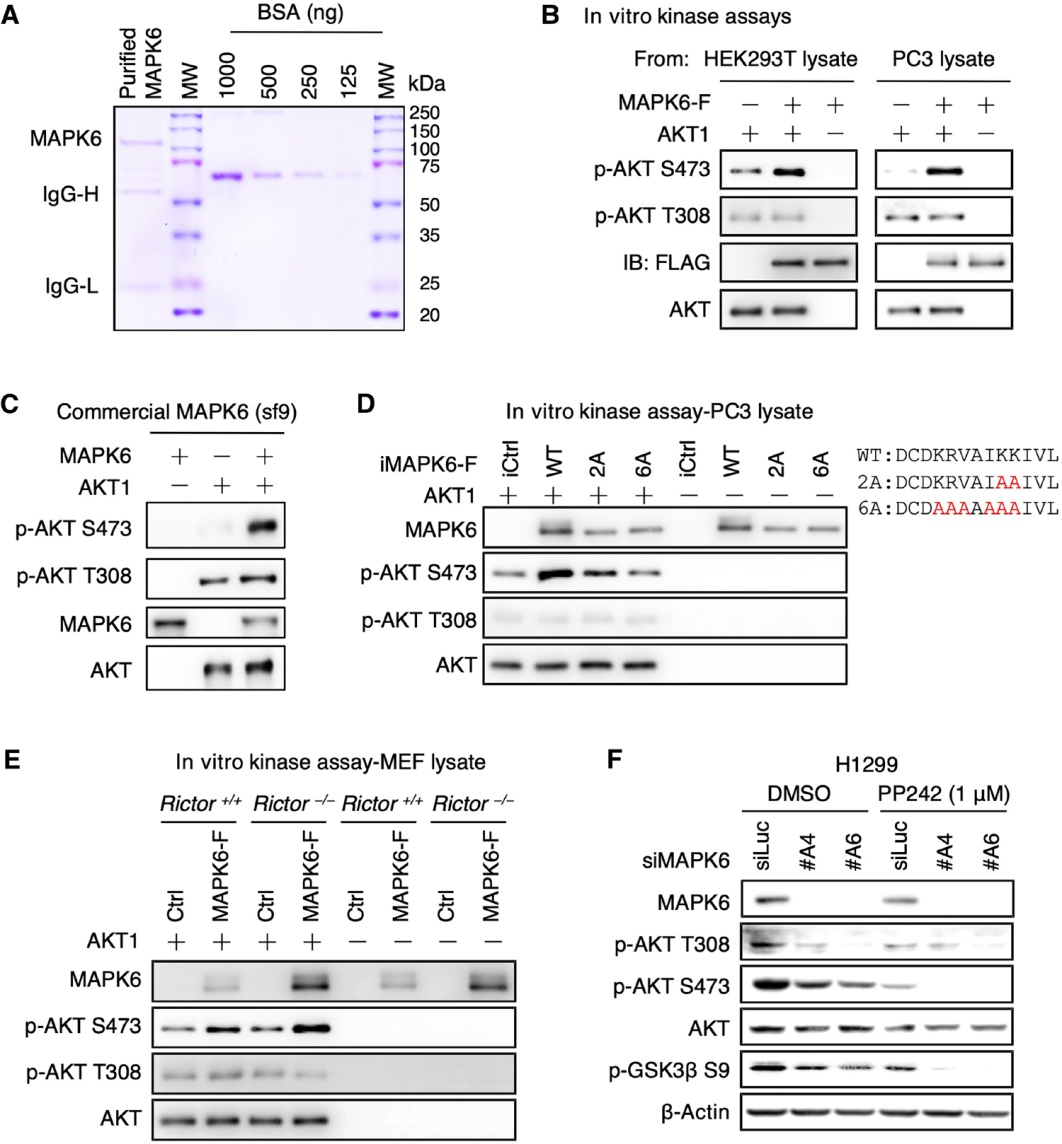
MAPK6 phosphorylates AKT at S473 independent of mTORC2. (**A**) Coomassie blue staining of the purified MAPK6 protein from HEK293T cells. MW, molecular weight; IgG-H/IgG-L, heavy/light chain of IgG. (**B**) Western blots on the products from in vitro kinase assays assessing affinity-purified MAPK6 phosphorylation of AKT1. MAPK6 proteins were overexpressed/purified using the EZview Red anti-FLAG M2 affinity gel from the HEK293T cells transiently transfected with pRK5-MAPK6-FLAG/His plasmid (left) or from the engineered PC3 cells overexpressing MAPK6-FLAG (right). Western blots on the products from in vitro kinase assays assessing AKT1 phosphorylation by (**C**) a commercially available MAPK6 protein, (**D**) wild-type (WT) MAPK6 versus MAPK6^2A^ versus MAPK6^6A^ mutants similarly expressed/purified from PC3 cells in (B), and (**E**) MAPK6-F (MAPK6-FLAG) proteins purified from the engineered *Rictor^+/+^* and *Rictor^−/−^* MEF cells with Dox-induced (0.5 μg/ml) expression of MAPK6-F or control (iCtrl). Also shown is the illustration of MAPK6^2A^ and MAPK6^6A^ mutants with indicated K/R to A mutations at the ATP binding pocket. (**F**) Western blots on H1299 cells transfected with siRNA targeting MAPK6 (siMAPK6 #A4, #A6) or control (siLuc) for 72 hours followed by 1 μM PP242 or DMSO treatments for 18 hours. Data are representative of at least three independent experiments.

The activation of AKT by MAPK6 could be either direct or indirect. To determine whether MAPK6 can directly bind and phosphorylate AKT, we affinity purified FLAG-tagged MAPK6 expressed in PC3 and HEK293T cells using anti-FLAG antibody–conjugated beads. The purified MAPK6 protein contained a major band of around 110 kDa, as detected by Coomassie blue staining (Fig. 5A). We then performed in vitro kinase assays using commercially available purified AKT1 protein as substrate, as previously described (*22*). MAPK6 protein, purified from either the HEK293T cells or the PC3 cells, strongly phosphorylated AKT1 on S473 but not T308 (Fig. 5B). Besides, a commercially available MAPK6 protein overexpressed/purified from sf9 cells also strongly phosphorylated AKT1 on S473 but not T308 in these in vitro kinase assays (Fig. 5C). To confirm that the kinase activity is required for MAPK6 phosphorylating AKT at S473, as illustrated in Fig. 5D, we made FLAG-tagged MAPK6^2A^ and MAPK6^6A^ mutants with disrupted VAIKK motif for adenosine triphosphate (ATP) binding (KRVAIKK at positions 44 to 50, to KRVAIAA and AAAAAAA, respectively). We similarly expressed and purified these proteins from PC3 cells and performed in vitro kinase assays. We observed that the MAPK6^2A^ mutant greatly inhibited and the MAPK6^6A^ mutant further blocked MAPK6-induced AKT phosphorylation at S473 (Fig. 5D). Together, these results support the role of MAPK6 as a novel AKT S473 kinase.

mTORC2, is a key AKT S473 kinase (*22*). Our in vitro kinase assay data suggest mTORC2-independent S473 phosphorylation of AKT by MAPK6. To further confirm this, we expressed and affinity purified MAPK6-FLAG proteins from *Rictor* knockout mouse embryonic fibroblasts (MEFs) (*23*) and wild-type MEFs using the anti-FLAG antibody–conjugated beads. As expected, purified MAPK6 from *Rictor* knockout MEFs maintained its ability to phosphorylate AKT1 S473 in vitro (Fig. 5E). Last, we treated MAPK6-knockdown H1299 cells and controls with PP242, an mTOR kinase–specific inhibitor (*24*). As expected for independent functions of the mTORC2 and MAPK6 pathways, knockdown of MAPK6 alone or PP242 treatment alone reduced AKT S473 phosphorylation, but cotreatment largely abolished it (Fig. 5F). Collectively, these results support MAPK6 phosphorylation of S473 independent of mTORC2.

### MAPK6 directly binds AKT

**Fig. 6.**
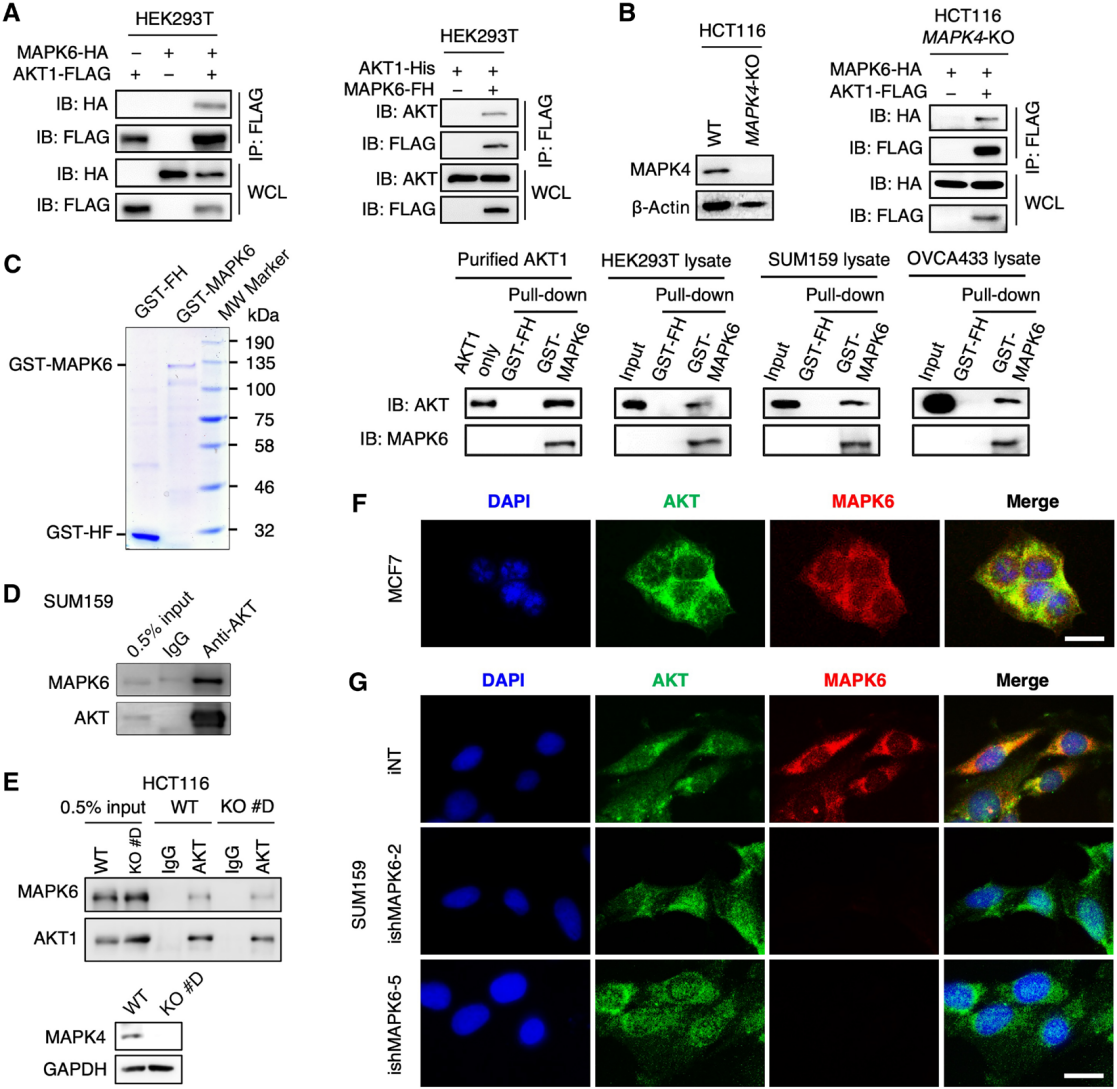
MAPK6 binds to AKT. (**A**) Western blots on immunoprecipitation products using anti-FLAG M2 affinity gel. HEK293T cells were transfected with MAPK6-HA/AKT1-FLAG (left) or AKT1-His/MAPK6-FH (Right). Forty-eight hours later, cell lysates were prepared and used for the studies. (**B**) Western blots on WT and *MAPK4*-KO HCT116 cell lysates (left) and coimmunoprecipitation of MAPK6-HA and AKT1-FLAG in the absence of MAPK4 (right). *MAPK4*-KO HCT116 cells were transfected with MAPK6-HA and AKT1-FLAG. Forty-eight hours later, cell lysates were prepared for immunoprecipitation using anti-FLAG M2 affinity gel and Western blots. (**C**) GST pull-down assays showing purified GST-MAPK6 binding with purified AKT1 and endogenous AKT in various cell lysates. Coomassie blue staining (left) revealed a major band of around 130 and 30 kDa in the purified GST-MAPK6 and GST-FH, respectively. MW, molecular weight. (**D** and **E**) Coimmunoprecipitation of endogenous MAPK6 with endogenous AKT from (D) SUM159 and (E) WT and *MAPK4-KO* HCT116 cell lysates. (**F** and **G**) Immunofluorescence staining showing colocalization of endogenous MAPK6 and AKT in (F) MCF7 and (G) SUM159 cell cytoplasm. Scale bars, 20 μm. The specificity of the MAPK6 antibody was confirmed by a lack of immunofluorescence signal in the MAPK6-knockdown (ishMAPK6-2, ishMAPK6-5) SUM159 cells. Data are representative of at least three independent experiments.

To further define the MAPK6-AKT pathway, we investigated MAPK6-AKT interaction. We expressed FLAG-tagged AKT1 in HEK293T cells overexpressing HA-tagged MAPK6 and performed immunoprecipitation using anti-FLAG antibody–conjugated beads followed by Western blot for HA. We also expressed a FLAG/His (FH)–tagged MAPK6 in HEK293T cells overexpressing a His-tagged AKT1 and performed immunoprecipitation using anti-FLAG antibody–conjugated beads followed by Western blot for AKT. In both cases, we readily detected MAPK6 and AKT1 interaction (Fig. 6A).

MAPK6 and MAPK4 can form a heterodimer (*25*), and we reported that MAPK4 could bind AKT (*19*), raising the prospect that MAPK4 could mediate apparent MAPK6-AKT interaction. To critically test this, we expressed FLAG-tagged AKT1 in *MAPK4*-KO HCT116 cells (*19*) overexpressing HA-tagged MAPK6 and performed immunoprecipitation using anti-FLAG antibody–conjugated beads followed by Western blot for HA. Again, we confirmed MAPK6-AKT interaction in the absence of MAPK4 (Fig. 6B). The direct interaction of MAPK6 and AKT was strongly confirmed by the demonstration that overexpressed and purified glutathione *S*-transferase (GST)–MAPK6 fusion protein, but not GST alone, can pull down commercially available purified AKT1 protein (Fig. 6C). Pull-down assays using HEK293T, SUM159, and OVCA433 cell lysates confirmed that GST-MAPK6 protein, but not GST, can pull down the endogenous AKT in these cell lysates (Fig. 6C). To confirm the physiological interaction between MAPK6 and AKT, we immunoprecipitated endogenous AKT from SUM159 cell lysate followed by Western blot for MAPK6. Again, we readily detected the interaction of endogenous MAPK6 and AKT (Fig. 6D). We further similarly examined the physiological interaction between MAPK6 and AKT in HCT116 cells and *MAPK4-KO* HCT116 cells. We confirmed coimmunoprecipitation and interaction of endogenous MAPK6 and AKT in both of these cells, indicating that MAPK6 can directly bind AKT independent of MAPK4 (Fig. 6E).

MAPK6 protein locates in both cytoplasm and nucleus (*16*). Since AKT primarily acts in the cytoplasm, we expected colocalization of cytoplasmic MAPK6 and AKT. Immunofluorescence using anti-MAPK6 and anti-AKT antibodies confirmed this cytoplasmic colocalization in MCF7 (Fig. 6F) and SUM159 cells (Fig. 6G). MAPK6 knockdown abolished this coimmunofluorescence (Fig. 6G). These data support the notion that MAPK6 binds to AKT in the cytoplasm for MAPK6-induced AKT activation.

### MAPK6 and MAPK4 bind AKT by different mechanisms

**Fig. 7.**
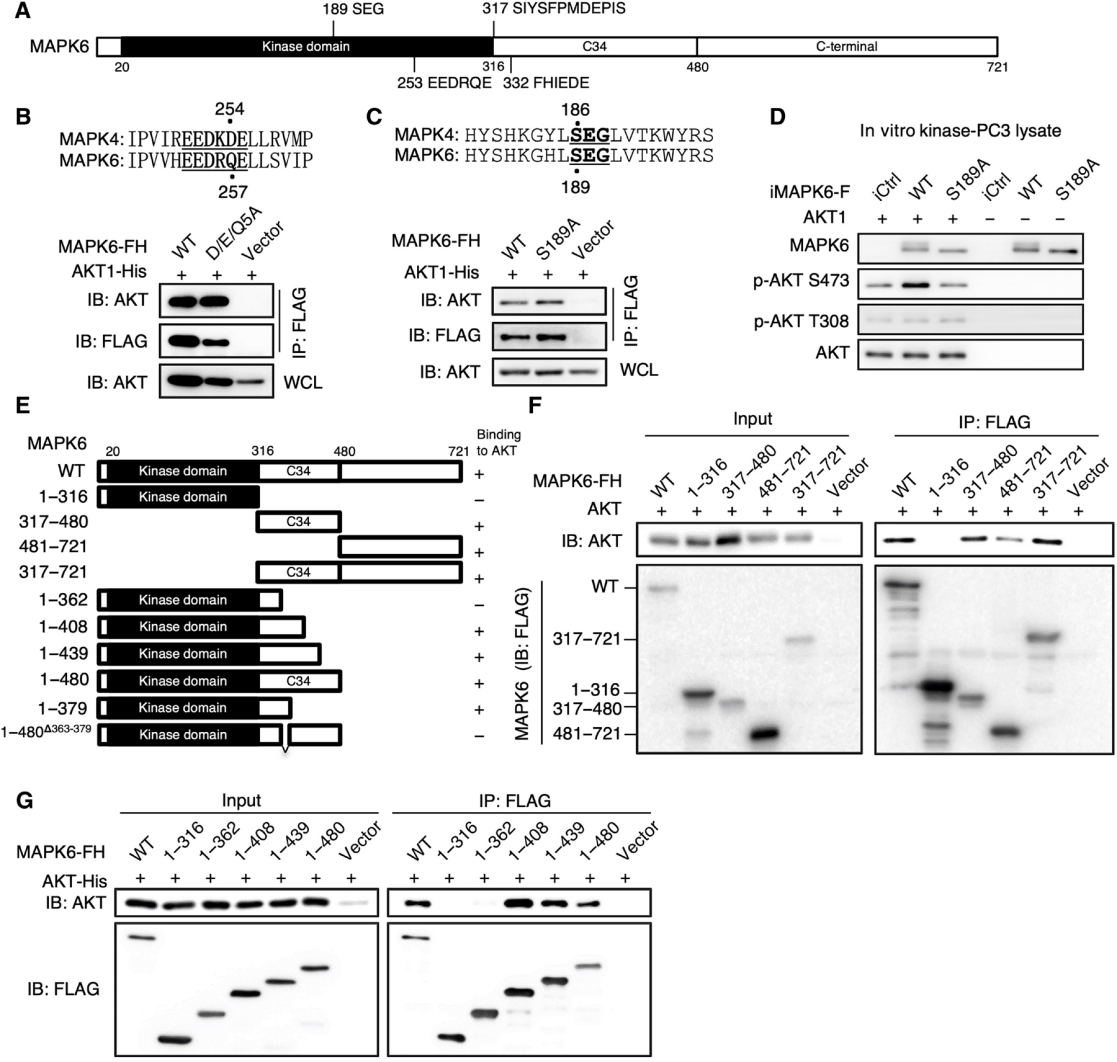
The molecular basis for MAPK6 binding to AKT, part 1. (**A**) Diagram for human MAPK6 domain architecture. MAPK6 contains an N-terminal kinase domain (20 to 316), a conserved C34 motif (317 to 480), and a C-terminal tail (481 to 721). Western blots on immunoprecipitation products for interaction between AKT1-His and the FH-tagged (**B**) WT versus a mutant (D/E/Q5A: EEDRQE to AAARAA) MAPK6, and (**C**) WT versus S189A mutated MAPK6. HEK293T cells were transfected with AKT1-His and FH-tagged WT or the indicated MAPK6 mutants. Forty-eight hours later, cell lysates were prepared for immunoprecipitation using anti-FLAG M2 affinity gel and Western blots. WCL, whole cell lysate. FH: 2× FLAG and 10× His tag. (**D**) In vitro kinase assays on AKT1 phosphorylation by WT versus S189A mutated MAPK6 proteins expressed/purified from engineered PC3 cells as similarly described in Fig. 5B. (**E**) Diagram for MAPK6 fragments/mutants used in (F) and (G) and Fig. 8 (C and D). (**F** and **G**) Western blots on the input and immunoprecipitation products for interaction between AKT1 and FH-tagged truncated MAPK6. HEK293T cells were similarly transfected/processed, as described in (B). Data are representative of at least three independent experiments.

MAPK6 contains an N-terminal kinase domain, a C34 conserved region shared between MAPK6 and MAPK4, and a unique long C-terminal tail (Fig. 7A). Since we previously identified a D/E-enriched common docking (CD) motif within MAPK4 (EEDKDE at positions 250 to 255) providing the critical docking site for AKT binding, we first investigated whether AKT binds to MAPK6 via the similar D/E-enriched motif (EEDRQE) at positions 253 to 258 of MAPK6 (Fig. 7B, top). For this purpose, we expressed a FLAG/His–tagged MAPK6 mutant with EEDRQE mutated to AAARAA (D/E/Q-5A) and His-tagged AKT1 in HEK293T cells and performed immunoprecipitation using anti-FLAG antibody–conjugated beads followed by Western blot for AKT. Mutation of these D/E/Q residues had minimal effect on MAPK6-AKT interaction (Fig. 7B), indicating that this putative CD motif is not required for MAPK6 binding to AKT.

Conventional MAP kinases share a conserved TXY motif in the activation loop that is the target for MAPKK-induced serine/threonine and tyrosine dual phosphorylation. In MAPK4 and MAPK6, this is replaced by SEG (S186 for MAPK4 and S189 for MAPK6; Fig. 7C, top). We have shown that S186A mutation inhibited MAPK4 binding and phosphorylation of AKT (*19*). To assess whether S189 also plays a similar role in MAPK6, we introduced His-tagged AKT1 and FLAG/His–tagged wild-type MAPK6 or the S189A mutant MAPK6 (MAPK6^S189A^) into HEK293T cells. We found that, unlike the MAPK4^S186A^ mutant, the MAPK6^S189A^ mutant maintained AKT1 interaction (Fig. 7C). However, the MAPK6^S189A^ mutant exhibited little activity in phosphorylating AKT1 S473 in the in vitro kinase assay (Fig. 7D).

Together, our data strongly suggest that MAPK6 uses a different mechanism to bind to AKT. This MAPK6-AKT binding is neither dependent on the previously identified D/E-enriched CD motif nor dependent on the SEG motif.

### C34 region provides the essential site for MAPK6 binding and phosphorylating AKT

To determine the critical region of MAPK6 required for AKT interaction, we performed coimmunoprecipitation of His-tagged AKT1 with FLAG/His-tagged fragments of MAPK6 overexpressed in HEK293T cells. In contrast to the MAPK4 kinase domain, which can efficiently bind AKT (*19*), the kinase domain of MAPK6 [the 1 to 316 amino acid (aa) fragment of MAPK6, MAPK6^1-316^] failed to interact with AKT (Fig. 7, E and F). Instead, the C34 region (the 317 to 480 aa fragment of MAPK6, MAPK6^317-480^), the C-terminal tail (MAPK6^481-721^), and the fragment containing both C34 and C-terminal tail (MAPK6^317-721^) were sufficient to bind AKT (Fig. 7, E and F). The C34 region appeared to exhibit a higher AKT binding affinity than that of the C-terminal tail (Fig. 7, E and F). These data suggest that MAPK6 binds to AKT through both the higher-affinity C34 region and the lower-affinity C-terminal tail.

To map the high-affinity site, we further created a series of C-terminally truncated MAPK6 containing either the intact or part of the C34 region (Fig. 7E). MAPK6 fragments MAPK6^1-480^, MAPK6^1-439^, and MAPK6^1-408^, but not MAPK6^1-362^ or MAPK6^1-316^, maintained high affinity to AKT (Fig. 7G). This suggests that the 362 to 408 region of MAPK6 contains a motif critically required for MAPK6-AKT interaction.

**Fig. 8.**
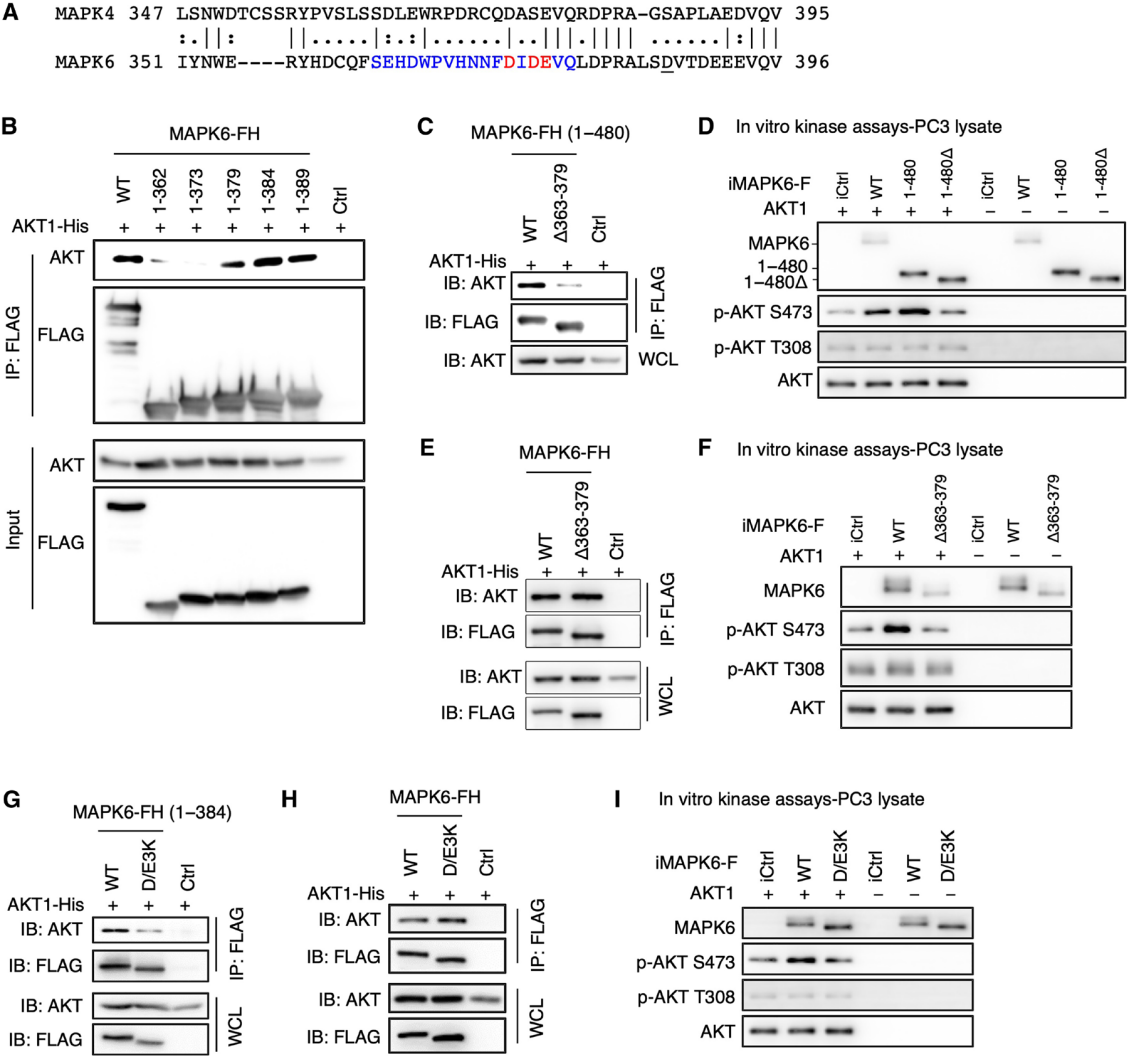
The molecular basis for MAPK6 binding to AKT, part 2. (**A**) Sequence alignment between MAPK6 and MAPK4 around the potential AKT binding motif of MAPK6. (**B** and **C**) Western blots on input and immunoprecipitation products for interaction between AKT1 and FH (2× FLAG and 10× His)–tagged truncated MAPK6. HEK293T cells were similarly transfected/processed, as described in Fig. 7B. MAPK6^1-379^ but not MAPK6^1-373^ can bind AKT (B). Deletion of 363 to 379 [Δ363–379, SEHDWPVHNNFDIDEVQ in blue/red, (A)] suppressed MAPK6^1-480^ interaction with AKT1 (C). (**D**) In vitro kinase assays on AKT1 phosphorylation by FLAG-tagged MAPK6, MAPK6^1-480^, and MAPK6^1-480Δ363-379^ proteins expressed/purified from PC3 cells, as similarly described in Fig. 5B. (**E** and **F**) MAPK6^Δ363-379^ maintained AKT1 binding but could not phosphorylate AKT1. FLAG-tagged MAPK6^Δ363379^ was expressed/purified, and assays were performed as similarly described in (C) and (D). (**G** and **H**) Western blots on immunoprecipitation products for AKT1 and MAPK6 mutant interactions. Mutation of DIDE [374 to 377, highlighted in red (A)] into KIKK (D/E3K) blocked MAPK6^1-384^ (G) but not full-length MAPK6 (H) binding to AKT1. (**I**) In vitro kinase assays on AKT1 phosphorylation by FLAG-tagged WT and D/E3K-mutated MAPK6 proteins expressed/purified from PC3 cells, as similarly described in Fig. 5B. WCL, whole cell lysate. Data are representative of at least three independent experiments.

To further map and define the critical region for MAPK6-AKT binding, we also generated additional C-terminally truncated fragments. MAPK6^1-379^, MAPK6^1-384^, and MAPK6^1-389^, but not MAPK6^1-373^ or MAPK6^1-362^, maintained their affinity to AKT (Fig. 8, A and B), identifying the 374 to 379 aa region of MAPK6 containing at least part of the sequence critical for AKT binding. In accord with their distinct AKT binding properties, MAPK6 and MAPK4 share little homology within this 374 to 379 or the broader 363 to 379 region (Fig. 8A and fig. S2, A and B).

Accordingly, the MAPK6^1-480^ mutant with an internal 363 to 379 deletion (MAPK6^1-480**Δ**363-379^) mostly lost AKT binding (Fig. 8C) despite maintaining MK5 binding through the FHIEDE motif (Fig. 7A and fig. S2C). Consistent with this, MAPK6^1-480^ exhibited kinase activity in phosphorylating AKT at S473 in the in vitro kinase assay, while MAPK6^1-480Δ363-379^ largely lost this activity (Fig. 8D). We hypothesized that a candidate D/E-enriched motif within the 363 to 379 aa region of MAPK6 (DIDE, 374 to 377 aa) might be a CD motif for MAPK6 and AKT interaction. In accord with this, mutation of DIDE to KIKK (D/E3K) abolished binding between MAPK6^1-384^ and AKT (Fig. 8G). In contrast, neither Δ363–379 nor D/E3K mutation affected binding between full-length MAPK6 and AKT (Fig. 8, E and H); however, they both potently blocked MAPK6 AKT kinase activity (Fig. 8, F and I). Collectively, these data suggest that the C-terminal tail of MAPK6 can provide an AKT binding site, which may underlie MAPK6^**Δ**363-379^ and MAPK6^D/E3K^ binding to AKT. However, the 363 to 379 region or DIDE motif is critically required for the proper docking of AKT onto MAPK6 for MAPK6 phosphorylating AKT.

### Inhibiting MAPK6 sensitizes cancer cells to mTOR kinase inhibitors

**Fig. 9.**
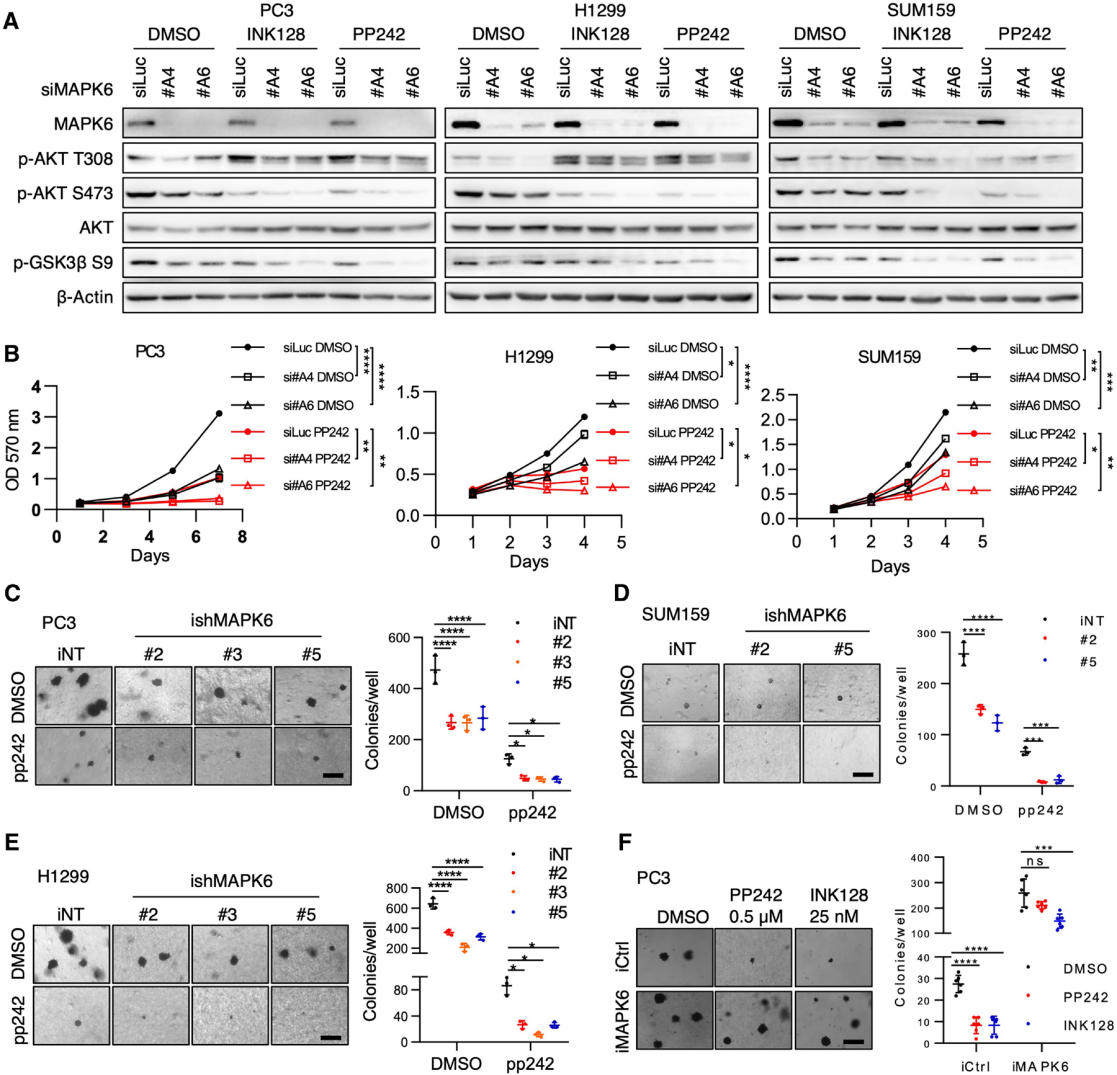
Knockdown of MAPK6 sensitizes cancer cells to mTOR kinase blockade. (**A**) Western blots and (**B**) proliferation assays on PC3, H1299, and SUM159 cells with siRNA-mediated knockdown of MAPK6 (siMAPK6 #A4, #A6) or luciferase control (siLuc) and treated with PP242 and/or INK128. Cells were first transfected with the indicated siRNA. Seventy-two hours later, cells were treated with PP242 (0.5 μM) or INK128 (25 nM) for 6 hours and processed for Western blots. For proliferation assays, 48 hours after transfection, cells were plated into 24-well plates and treated with PP242 (1 μM for PC3 and SUM159, and 0.5 μM for H1299 cells) or DMSO. (**C** to **E**) Soft-agar assays on (C) PC3, (D) SUM159, and (E) H1299 cells with Dox-induced (1 μg/ml) knockdown of MAPK6 (ishRNA #2, #3, and/or #5) or control (iNT) treated with PP242 (concentrations as described above) or DMSO. (**F**) Soft-agar assays on Dox-induced (1 μg/ml) PC3-iMAPK6 and iCtrl cells treated with 0.5 μM PP242, 25 nM INK128, or DMSO. Data as means ± SD, two-way ANOVA with multiple comparisons corrected with Sidak’s test. *P ≤ 0.05. **P ≤ 0.01. ***P ≤ 0.001. ****P ≤ 0.0001. ns, not significant. Data are representative of at least three independent experiments.

Despite the promise of mTOR kinase inhibitors, they only exhibited limited activities in cancer clinical trials. We identified MAPK6 as an S473 kinase of AKT, independent of mTORC2 (Fig. 5). This raised the question of whether MAPK6 affects cancer cell sensitivity to mTOR kinase inhibition. PP242 is one of the first identified mTOR kinase inhibitors (*24*), and INK128/sapanisertib (*26*) is a potent and highly specific mTOR kinase inhibitor being tested in cancer clinical trials. Therefore, we examined how MAPK6 affects PP242 and INK128 activities in repressing cancer cell growth. While knockdown of MAPK6 (siRNA in proliferation assay and shRNA in soft-agar assays) or treatments using PP242 or INK128 each repressed AKT phosphorylation and PC3, H1299, and SUM159 cancer cell growth, including anchorage-independent growth, treatment of both achieved the most robust activity (Fig. 9, A to E, and fig. S3, A to D). These data supported that knockdown of MAPK6-sensitized cancer cells to mTOR kinase inhibition. Accordingly, MAPK6 overexpression both promoted the anchorage-independent growth of PC3 cells and rendered them resistant to PP242 and INK128 treatments (Fig. 9F). Collectively, these data supported that MAPK6 and mTORC2 independently and coordinately promote AKT phosphorylation/activation and that MAPK6 emerges as a key regulator for cancer cells’ response to mTOR kinase blockade.

### MAPK6 overexpression correlates with poor survival of patients with cancer

**Fig. 10.**
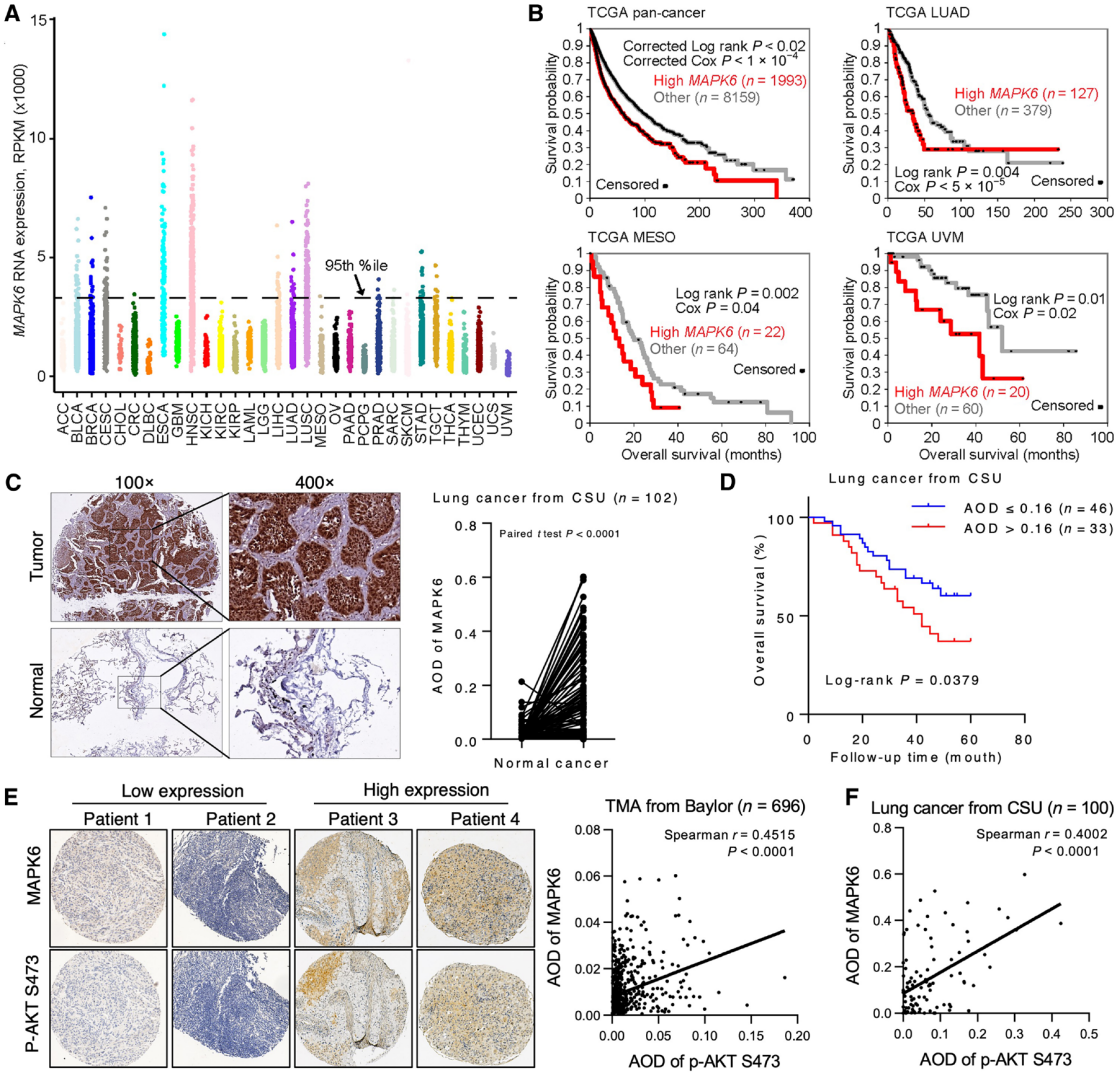
High MAPK6 expression is associated with decreased overall survival and increased AKT phosphorylation. (**A**) MAPK6 mRNA expression across 10,152 tumors from TCGA. (**B**) Kaplan-Meier plots of overall survival, both across all cancer types in TCGA cohort (“pan-cancer”) and within specific cancer types as indicated, as stratified by high MAPK6 expression. *P* values by univariate Cox or log-rank test (stratified tests correcting for overall differences in survival according to tumor type for pan-cancer plot). (**C**) Representative MAPK6 IHC staining on a human lung cancer TMA consisting of *n* = 102 matched normal and cancer tissues from CSU. Also shown are paired comparisons of AOD of tumor versus normal lung tissue MAPK6 staining. *P* values by paired *t* test. (**D**) Kaplan-Meier plots of overall survival in the CSU cohort with available follow-up data. Mean AOD of 1.6 was used to divide patients into MAPK6-high and MAPK6-low groups. *P* values by stratified log-rank test. (**E**) Representative MAPK6 and phospho-AKT S473 staining on a pan-cancer TMA from Baylor. Analysis of (**E**) *n* = 696 Baylor pan-cancer and (**F**) *n* = 100 CSU lung cancer tissues with available MAPK6/phospho-AKT readouts revealed positive correlations of MAPK6 expression and AKT phosphorylation in both (Spearman’s correlation coefficient).

Our data support an oncogenic and tumor-promoting activity of MAPK6 that predicts a negative correlation between *MAPK6* expression and cancer patient survival. A comprehensive analysis of gene expression profiles of 10,152 patients in The Cancer Genome Atlas (TCGA) (*27*) revealed that *MAPK6* is expressed at varying levels in all cancer types (Fig. 10A). In this large pan-cancer panel, survival was significantly decreased in the subset of patients above the 80th percentile of *MAPK6* expression (Fig. 10B). Although shorter follow-up data and small numbers of patients limited analysis in some cases, survival was also markedly decreased in patients with *MAPK6* overexpression above the 75th percentile in several specific cancer types in the TCGA dataset, including lung adenocarcinoma (LUAD), mesothelioma (MESO), and uveal melanoma (UVM). Analysis of the cancer compendium also showed decreased survival in patients with LUAD (*28*) and patients with breast cancer (*29*) with *MAPK6* overexpression (fig. S4A).

We further assessed MAPK6 protein expression in a human lung cancer tissue microarray (TMA) consisting of *n* = 102 matched normal and cancer tissues from the Xiangya Hospital of Central South University, China (*n* = 50 LUAD, *n* = 49 squamous cell lung cancer, and *n* = 3 adenosquamous carcinoma). We observed that MAPK6 protein expression was elevated in cancer tissues (Fig. 10C and fig. S4B) and that MAPK6 high expression correlated with a markedly decreased overall survival (Fig. 10D). MAPK6 high expression also associated with tumor stage (T3–4) and differentiation (G3–4), but not tumor pathological type, N stage, or patient age or gender (fig. S4C). Collectively, these data support a tumor-promoting role of MAPK6.

### MAPK6 protein expression correlates with AKT phosphorylation

To further examine the identified MAPK6-AKT axis in human cancers, we also assessed MAPK6 protein expression and AKT phosphorylation in a pan-cancer TMA from Baylor College of Medicine and the above human lung cancer TMA from Xiangya Hospital. As our identified MAPK6-AKT signaling axis predicts, MAPK6 protein expression correlated with AKT phosphorylation in both these cancer TMAs, which further supports the MAPK6-AKT signaling axis in human cancers (Fig. 10, E and F, and fig. S4D).

## DISCUSSION

The limited numbers of previous studies on MAPK6 biology in human cancers have focused on regulation of the cancer cell migration/invasion/metastasis (*8*, *10*–*15*). MAPK6 also promoted cervical cancer cell growth, but inhibited melanoma and intrahepatic cholangiocarcinoma cell proliferation (*10*, *13*, *14*, *17*, *18*). To date, it appears that whether MAPK6 executes tumor-promoting or tumor-suppressing function is cancer type or cancer cell context dependent. To critically examine how MAPK6 regulates human cancers, we used six common human cancer cell lines representing four different cancer types, including prostate cancer (PC3 and DU145), breast cancer (MCF7 and SUM159), ovarian cancer (OVCA433), and non–small cell lung cancer (H1299). We also assessed the oncogenic activities of MAPK6 using two immortalized normal human epithelial cell lines: the prostate epithelial cell line PNT1A and the breast epithelial cell line MCF10A. We demonstrated that MAPK6 overexpression transforms both human epithelial cell lines into anchorage-independent growth and also promotes growth, including anchorage-independent growth of all five cancer cell lines tested (PC3, DU145, SUM159, H1299, and OVCA433). These data clearly define the potent oncogenic and tumor-promoting activities of ectopically overexpressed MAPK6 in all the seven cell lines examined. Accordingly, knockdown of MAPK6 in four cancer cell lines with medium to high expression of endogenous MAPK6 (MCF7 and SUM159 for breast cancer, PC3 for prostate cancer, and H1299 for non–small cell lung cancer) inhibited cell growth in vitro and/or xenograft growth in vivo.

We recently reported that MAPK4, another atypical MAPK most closely related to MAPK6, promotes cancer by noncanonically activating the key oncogenic kinase AKT independent of PI3K/PDK1 (*19*). MAPK4 acts as a T308 kinase and also induces mTORC2-dependent S473 phosphorylation. In contrast, MAPK6 mainly functions as an S473 kinase in the in vitro kinase assay, independent of mTORC2 (Fig. 5, B to E). MAPK6 also induces AKT phosphorylation at T308 in cells, indicating the potential involvement of PI3K/PDK1 and/or MAPK4 for this phosphorylation. When ectopically overexpressed in HEK293T cells, MAPK6 appeared to have more robust activities than MAPK4 in phosphorylating AKT—the HA-tagged MAPK6 exhibited similar activities in phosphorylating AKT when expressed at a substantially lower level than that of the HA-tagged MAPK4. Ectopic overexpression of MAPK6 in eight tested cell lines, including PC3, DU145, SUM159, OVCA433, H1299, PNT1A, MCF10A, and HEK293T cells, induced AKT phosphorylation/activation. In accord with this, knockdown of endogenous MAPK6 repressed AKT phosphorylation and activation in the four tested cell lines (MCF7, PC3, SUM159, and H1299) with high to median endogenous MAPK6 expression, further confirming the MAPK6-AKT pathway in the cells. Furthermore, knockdown of MAPK6 greatly inhibited AKT phosphorylation and H1299 xenograft tumor growth, and overexpression of MAPK6 promoted AKT phosphorylation and PC3 xenograft tumors, demonstrating the tumor-promoting MAPK6-AKT pathway in vivo. Last, we observed that MAPK6 protein expression correlated with AKT phosphorylation in both a pan-cancer (Baylor) cohort and a lung cancer [Central South University, China (CSU)] cohort. Collectively, these strongly support our identified MAPK6-AKT pathway in human cancers.

Human MAPK4 and MAPK6 share a similar domain structure (*1*). We have previously reported that a CD motif within the kinase domain of MAPK4 mediates MAPK4-AKT binding, and the D254 within the CD motif is essential for this interaction. Besides, phosphorylation of S186 of the SEG motif also appears to play a critical role in regulating MAPK4-AKT interaction. D254 of MAPK4 is replaced by Q257 in the corresponding potential CD motif of MAPK6, suggesting that MAPK6 may use a different mechanism for AKT binding. Neither disruption of the potential CD motif around Q257 nor mutation of S189 (corresponding to S186 in MAPK4) affected MAPK6-AKT interaction. In accord with this, the kinase domain of MAPK6, unlike that of MAPK4, lacks binding affinity to AKT. Other than the D254/Q257 variation, the CD motifs of MAPK4 and MAPK6 within the kinase domain are highly conserved. A Q257D mutation or an MM mutation with the entire CD motif and the flanking sequence replaced by the corresponding sequence of MAPK4 largely restored MAPK6 kinase domain binding with AKT, confirming the potential functionality of this largely conserved CD motif in MAPK6 (fig. S5). This further confirms, in a different context, the essential role of D254 in MAPK4-AKT binding.

MAPK4 and MAPK6 share relatively high homology within the C34 region. We identified that the SEHDWPVHNNFDIDEVQ fragment at 363 to 379 aa of the MAPK6 C34 region is critical for MAPK6^1-480^ binding to AKT. This sequence is not conserved in MAPK4, which may underlie its unique ability to bind AKT. The unique long C-terminal tail of MAPK6 also contains an AKT binding activity with somewhat lower apparent affinity. All in all, in contrast to the MAPK4 kinase domain–mediated binding of AKT, MAPK6 uses a distinct mechanism based on unique sequences within C34 and the C-terminal tail.

AKT activity was required for MAPK6 oncogenic and tumor-promoting activities under the conditions that we examined. Since both the canonical PI3K/PDK1 pathway and our recently identified MAPK4 pathway can independently regulate AKT activation, the tumor-promoting activities of endogenous MAPK6 are likely to be context dependent. Because MAPK6 has been reported in both the cytoplasm and the nucleus, it is expected that activation of cytoplasmic AKT will not be substantial in cancer cells where MAPK6 mainly locates in the nucleus. Our results highlight the importance of understanding the molecular mechanism underlying MAPK6 cytoplasm-nucleus translocation and its functional significance.

MAPK6 appears to function as an S473 kinase of AKT, independent of mTORC2 (Fig. 5). While inhibiting MAPK6 (knockdown) and mTOR kinase activity (treatments using PP242 or INK128) each represses AKT phosphorylation and cancer cell growth, including anchorage-independent growth, treatment of both achieves the robust activity (Fig. 9 and fig. S3). This supports the notion that MAPK6 and mTORC2 independently and coordinately phosphorylate AKT at S473 and indirectly at T308 for tumor-promoting effects. The pro-oncogenic role of MAPK6 described here is also strongly supported by the observation that elevated *MAPK6* mRNA expression negatively correlates with pan-cancer patient survival and survival in specific cancer types, including LUAD, MESO, UVM, and breast cancer. This raises the prospect that blocking MAPK6, either alone or combined with mTOR inhibitors, will be an effective therapeutic for MAPK6-high cancers. Our study warrants future works to develop MAPK6-specific inhibitors and test their efficacy in treating human cancers, especially in combination therapy, such as that in combination with mTOR kinase inhibition.

## MATERIALS AND METHODS

### Plasmids

The pInducer20-YF vector was generated as previously described (*19*). pRK5 vector was provided by X.-H. Feng at Baylor College of Medicine, Houston, Texas. The CDS (CoDing Sequence) of *MAPK6* was polymerase chain reaction (PCR) amplified from the LNCaP cell cDNA, and sequencing verified to contain the same sequence as CDS in NM_002748 in GenBank. The CDS of *MAPK6* was cloned into the pInducer20-YF vector between the Mlu I and Sal I sites to produce the pInducer-MAPK6 vector. To generate the pRK5-MAPK6-FH (2× Flag and 10× His) vector, we first subcloned the MAPK4-FH CDS from the pCDH-MAPK4-FH vector (*19*) into the pRK5 vector between Cla I and Not I. We then replaced the *MAPK4* CDS between MIu I and Pac I in the pRK5-MAPK4-FH vector with the full-length, truncated, and mutated CDS of *MAPK6.* These generated the pRK5-MAPK6-FH vector along with those containing the truncated and mutated MAPK6. We also subcloned the CDS of MAPK6^1-480^ from the pRK5-MAPK6^1-480^-FH vector into the pInducer20-YF between the Mlu I and Xho I sites to produce the pInducer-MAPK6^1-480^-FH vector. We used a similar strategy to generate the pInducer-MAPK6^1-480Δ363-379^-FH vector. Vectors containing AKT1 were reported previously (*19*). Table S1 contains primers used for cloning wild-type, mutant, and truncated MAPK6. We used MAPK6^2A^ as the template to generate MAPK6^6A^.

### Cell culture, transient transfection, and lentivirus infection

MEF *Rictor^+/+^* and MEF *Rictor^−/−^* cells were obtained from M. Magnuson at Vanderbilt University, Nashville, Tennessee (*23*). *MAPK4-KO* HCT116 cells were described previously (*19*). PNT1A cells were acquired from the European Collection of Authenticated Cell Cultures. Other cell lines were obtained from the American Type Culture Collection. MCF10A cells were cultured in Dulbecco’s modified Eagle’s medium (DMEM) supplemented with 5% horse serum (#16050122, Gibco), hydrocortisone (0.5 μg/ml; #H-0888, Sigma-Aldrich), human epidermal growth factor (20 ng/ml; #E9644, Sigma-Aldrich), insulin (10 μg/ml; #CAS11061-68-0, Santa Cruz Biotechnology), cholera toxin (100 ng/ml; #C-8052, Sigma-Aldrich), penicillin (100 U/ml), and streptomycin (100 μg/ml). MCF7 cells were cultured in DMEM with insulin (10 μg/ml); HCT116, PNT1A, PC3 cells in RPMI 1640; and other cell lines in DMEM, all supplemented with 10% fetal bovine serum, penicillin (100 U/ml), and streptomycin (100 μg/ml). Transient transfection and lentivirus packaging/infection were done as we previously reported (*19*).

### shRNAs and siRNAs for knockdown

Lentiviral shRNA constructs were obtained from Open Biosystem (Thermo Fisher Scientific). Targeting sequences between Mlu I and Xho I sites were released from pGIPZ vectors and cloned into pInducer10 using the same sites. The targeting sequences are as follows: pGIPZ-shMAPK6 #2: AGAGTGTCAAACATGCTCT, pGIPZ-shMAPK6 #3: GTCATCATCATATTTAGAT, and pGIPZ-shMAPK6 #5: TGGGTCACCACTTAAGTCA. siRNAs were obtained from Invitrogen as follows: siMAPK6 #A4: GAGCUCUGUCCGAUGUCACUGAUGA and siMAPK6 #A6: GCCUUGUUGGCAAUACUCAGAUCAU.

### Proliferation assay

Cells were seeded at 5000 to 20,000 cells per well (cell line dependent) in 500 μl of media in 24-well plates. Cell cultures were stopped at predetermined time points, fixed with 10% (w/v) formaldehyde for 15 min, and stained with 0.05% (w/v) crystal violet in 10% ethanol and 10% methanol for 20 min at room temperature. After wash and air dry, 300 μl 10% acetic acid was added to each well, and absorbance was read at 570 nm. Dox was used for inducing gene overexpression (1 μg/ml) or knockdown (4 μg/ml). For proliferation assays on cells with siRNA-mediated knockdown of MAPK6, cells were first transfected with 10 nM siRNA. Forty-eight hours later, cells were plated in 24-well plates and used in proliferation assays. When applicable, mTOR kinase inhibitors PP242 (#S2218, Selleckchem) and INK128 (#HY-13328, MedChemExpress) were added to culture media the day after cell seeding.

### Plate colony formation assay and soft-agar assay

For plate colony assay, 500 to 1000 cells per well (cell line dependent) were seeded in six-well plates in 2 ml of media and resupplied with fresh media every week. After 2 to 3 weeks, cells were fixed with 10% (w/v) formaldehyde for 15 min and stained with 0.05% (w/v) crystal violet supplemented with 10% ethanol and 10% methanol for 20 min at room temperature. The plates were then washed with distilled water, air dried, and scanned using a Canon scanner. The cell colony numbers were then manually counted. Soft-agar assays were performed as previously described (*19*). When applicable, AKT inhibitors MK2206 (#S1078, Selleckchem) and GSK1411795 (#S7492, Selleck), and mTOR kinase inhibitors PP242 (#S2218, Selleckchem) and INK128 (#HY-13328, MedChemExpress) were added during setup and added with fresh media every week.

### Western blotting

Protein samples were prepared in radioimmunoprecipitation assay (RIPA) buffer [50 mM tris-HCl (pH 8.0), 100 mM NaCl, 0.5% sodium deoxycholate, 0.1% SDS, 10 mM NaF, 20 mM Na_3_VO_4_, 1% Triton X-100, and the protease inhibitors cocktail solution P3100-005 from Gendepot] and quantified using a bicinchoninic acid protein assay kit (#23225, Thermo Fisher Scientific). The samples were mixed with 6× loading dye and boiled at 97°C for 5 min. An equal amount of 10 to 20 μg protein per lane was used in SDS–polyacrylamide gel electrophoresis and immunoblotting analysis. Antibodies against p-AKT T308 (#13038 and #9275), p-AKT S473 (#4060), AKT (#9272), p-GSK3β S9 (#9336), RICTOR (#2114), HA-tag (#3724), and MK5 (#7419) were from Cell Signaling Technology (CST). Other antibodies used include anti-MAPK6 (#ab53277, Abcam), anti–β-Actin (#AC026, Abclonal), anti-FLAG (#200474, Agilent), and anti-MAPK4 (#AP7298b, Abcepta).

### In vitro kinase assay

The 10× reaction buffer [500 mM tris-HCl (pH 7.5), 1 mM EDTA, 2 mM NaCl, and 1% β-mercaptoethanol] and 5× Mg^2+^/ATP buffer (50 mM magnesium acetate and 2.5 mM ATP) were freshly prepared, stored at 4°C if needed, and used within 24 hours. Transient-transfected HEK293T cells (1 × 10^6^) or stable-infected PC3 cells (3 × 10^6^) both overexpressing FLAG-tagged MAPK6 protein were collected and inoculated in 1 ml of cell lysis buffer [20 mM tris-HCl (pH 7.5), 150 mM NaCl, 2 mM EDTA, 1% Triton X-100, 1 mM NaF, 1 mM Na_3_VO_4_, and 1× proteinase inhibitor cocktail] at 4°C for 30 min. The cell lysates were then centrifuged at 4°C at 13,800*g* for 10 min. The supernatant was mixed with EZview Red anti-FLAG M2 affinity gel (Millipore Sigma) and rotated for 90 min at 4°C. The anti-FLAG M2 affinity gel was then washed with lysis buffer twice and with 1× reaction buffer three times. The reaction system contains 3 μl of 10× reaction buffer, about 50 ng of MAPK6 protein, 6 μl of 5× Mg^2+^/ATP buffer, and 100 ng of recombinant His-AKT1 (inactive; #14-279, Millipore Sigma) in a final volume of 30 μl. Reaction conditions include incubation in a thermomixer (Eppendorf, 300 rpm) at 30°C for 1 hour, tapping tubes every 10 min, and final incubation at 37°C for 20 min. We also used a similar protocol to examine the activity of a commercially available MAPK6 protein (100 ng; #ab217835 from Abcam) to phosphorylate AKT1.

### GST pull down

The CDS of *MAPK6* was PCR amplified and cloned into the GTK3XF vector between EcoRI and Sal I sites to generate the GST-MAPK6 fusion using the following primers: forward, 5′-AAGGAATTCATGGCAGAGAAATTTGAAAGTCTCATG-3′; reverse, 5′- AAGGTCGACTTAGTTCAGATGTTTCAGAATGCTGCTGTATG-3′. The GST-MAPK6 fusion protein was purified and subjected to pull-down assay as we previously reported (*19*). GST protein was used as a control.

### Immunoprecipitation

Immunoprecipitation was performed using EZview Red anti-FLAG M2 affinity gel (Millipore Sigma), as we previously reported (*19*). To detect endogenous MAPK6-AKT interaction, SUM159, HCT116, and *MAPK4-KO* (#D) HCT116 cells were treated with 10 μM MG132 for 4 hours. Before harvest, cells were treated with insulin (10 μg/ml) for 15 min. Cell lysates were prepared in 10% glycerol buffer [20 mM tris-HCl (pH 8.0), 137 mM NaCl, 10% glycerol, 0.5% sodium deoxycholate, 0.5% Triton-X, and 0.5 mM MgCl_2_]. Immunoprecipitation was performed using anti-AKT antibody (#9272, CST), with normal rabbit immunoglobulin G (IgG; #2729, CST) as control. Anti-MAPK6 antibody (#ab53277, Abcam) and mouse anti-rabbit IgG antibody (Light-Chain Specific, L57A3, #3677 CST) were used to detect AKT-bound MAPK6. Mouse anti-AKT antibody (#2920, CST) was used to detect the immunoprecipitated AKT.

### Immunofluorescence

Cells were seeded on glass coverslips for 24 to 48 hours. Cells were then fixed with methanol for 15 min at 4°C and washed with phosphate-buffered saline (PBS) three times. Triton X-100 in PBS buffer (0.3%) containing 10% goat serum was used to increase nuclear membrane permeability and block nonspecific binding. The slides were then incubated with anti-AKT (1:100; #2920, CST) and anti-MAPK6 (1:100; #ab53277, Abcam) antibodies at 4°C overnight, followed by incubations with DyLight-488 goat anti-mouse IgG (1:500; #35502, Invitrogen) and Alexa Fluor 594 goat anti-rabbit IgG (1:500; #8889, CST) at room temperature for 1 hour. Last, the coverslips were stained with 4′,6-diamidino-2-phenylindole (DAPI), mounted onto glass slides with antifade mounting medium (#H-1000, Vector Laboratories), and examined/photographed under an Olympus microscope.

### Information of human samples

We used two sets of previously made TMA here. The first set is a pan-cancer TMA obtained from the Human Tissue Acquisition and Pathology core at Baylor College of Medicine. The exempt materials were collected and coded under the Baylor Institutional Review Board–approved protocols H-18890 and H-21543.

The second set is a lung cancer TMA previously built under protocol no. 201303177 approved by the Ethics Committee of Xiangya Hospital of CSU, China. Also used for Western blot assays were four pairs of lung cancer and normal tissues freshly collected under the same CSU protocol no. 201303177. All patients provided written informed consent and understood that their tissues would be used for research.

The Baylor TMA contains pan-cancer tissue samples [*n* = 696 available for immunohistochemistry (IHC) readouts with defined pathology] from both primary cancers (*n* = 435, main types include cancers of adrenal, *n* = 13; brain, *n* = 19; cervix, *n* = 20; endometrial, *n* = 21; esophagus/stomach, *n* = 15; kidney, *n* = 10; liver, *n* = 16; lung, *n* = 20; meninges, *n* = 18; ovary, *n* = 49; pancreas, *n* = 13; parotid gland, *n* = 17; pleura, *n* = 13; stomach, *n* = 22; testes, *n* = 15; thymus, *n* = 15; thyroid, *n* = 19; etc.) and metastatic cancers (*n* = 261; mainly from metastases to lymph nodes, *n* = 131;liver, *n* = 29; brain, *n* = 27; lung, *n* = 12; etc.).

The CSU TMA contains *n =* 102 paired cancer versus normal tissue (more than 10 cm from the tumor edge) samples collected from April 2014 to January 2018, including 49 squamous cell lung cancer, 50 LUAD, and 3 adenosquamous carcinoma of the lung. The survival data are available in 79 patients in this CSU cohort.

### Immunohistochemistry

Sample slides with 3-μm-thick tissue sections were deparaffined in xylene, rehydrated in descending concentrations of ethanol and PBS, and subjected to antigen retrieval by heated citrate buffer (pH 6.0). Endogenous peroxidases activities were blocked by 3% hydrogen peroxide. Slides were then incubated at 4°C overnight in the indicated antibodies, including those against MAPK6 (1:200; ab53277 from Abcam or sc-365234 from Santa Cruz Biotechnology) and p-AKT S473 (1:50, #3787 from CST or 1:150, AF11237 from AiFang Biological, China). IHC staining was then performed as previously described (*30*). IHC images were obtained on a Leica digital pathology slide scanner or a Leica microscope. IHC staining signal was evaluated on the basis of average optical density (AOD).

### Xenograft

H1299 cells (1 × 10^6^) and PC3 (3 × 10^6^) cells were subcutaneously injected into the lateral flanks of severe combined immunodeficient (SCID) mice (6 to 8 weeks old from Envigo) without Matrigel, while SUM159 cells (2 × 10^6^) were injected into the fourth mammary fat pad of SCID mice with Matrigel (1:1). Mice began receiving Dox (4 mg/ml for inducible knockdown and 0.5 mg/ml for inducible overexpression) in 1 to 5% sucrose in drinking water on the day of tumor inoculation. Tumors were monitored every 2 to 3 days, and tumor volumes were calculated and recorded as 0.52 × length × width^2^. Tumors were harvested as indicated and weighed. Part of the tumor was used for protein extraction with RIPA buffer and subjected to Western blots analysis. All animal studies were approved by the Institutional Animal Care and Use Committee of Baylor College of Medicine.

### Analysis of human tumor molecular datasets

For pan-cancer survival analyses, we collected molecular data on 10,152 tumors of various histological subtypes (ACC project, *n* = 79; BLCA, *n* = 407; BRCA, *n* = 1094; CESC, *n* = 304; CHOL, *n* = 36; CRC, *n* = 619; DLBC, *n* = 48; ESCA, *n* = 184; GBM, *n* = 159; HNSC, *n* = 519; KICH, *n* = 65; KIRC, *n* = 533; KIRP, *n* = 289; LAML, *n* = 163; LGG, *n* = 514; LIHC, *n* = 370; LUAD, *n* = 506; LUSC, *n* = 495; MESO, *n* = 86; OV, *n* = 261; PAAD, *n* = 178; PCPG, *n* = 179; PRAD, *n* = 497; SARC, *n* = 259; SKCM, *n* = 460; STAD, *n* = 411; TGCT, *n* = 134; THCA, *n* = 503; THYM, *n* = 119; UCEC, *n* = 544; UCS, *n* = 57; and UVM, *n* = 80) from TCGA, for which both RNA sequencing data (v2 platform) and patient survival data were available. Survival data were current as of 31 March 2016. Furthermore, we analyzed a compendium of LUAD expression profiles (*28*) and a compendium of breast cancer expression profiles (*29*) compiled previously. Breast cancer survival data were capped at 15 years. The log-rank test evaluated the top 20% (pan cancer) and top 25% (specific cancer type) of MAPK6-expressing cases versus the other cases, while univariate Cox evaluated the log_2_ MAPK6 expression as a continuous variable.

### Statistics

Unpaired two-tailed Student’s *t* test was used to analyze the statistical significance in the cell culture studies, and paired two-tailed Student’s *t* test was used for comparing xenograft tumor weights. The proliferation assay and in vivo xenograft growth were analyzed using two-way analysis of variance (ANOVA). Two-tailed tests and an α of 0.05 were used for all statistical analyses. Survival analysis was carried out using univariate Cox and log-rank test (stratified univariate Cox and stratified log-rank test for pan-cancer analysis, correcting for tumor type). The correlation between MAPK6 protein expression and AKT phosphorylation in human cancer TMA was determined by Spearman test.

## Notes

View/request a protocol for this paper from *Bio-protocol*.
