# Supplementary material for: MAPK6-AKT signaling promotes tumor growth and resistance to mTOR kinase blockade
Source: Sci Adv. 2021 Nov 12;7(46):eabi6439. doi: 10.1126/sciadv.abi6439 (PMC8589317; doi:10.1126/sciadv.abi6439)
Supplement: Supplementary file 1 — Table S1 Figs. S1 to S5 [file sciadv.abi6439_sm.pdf]

Science Advances
AAAS

Supplementary Materials for

MAPK6-AKT signaling promotes tumor growth and resistance to mTOR kinase blockade

Qinbo Cai, Wolong Zhou, Wei Wang, Bingning Dong, Dong Han, Tao Shen, Chad J. Creighton, David D. Moore, Feng Yang*

*Corresponding author. Email: fyang@bcm.edu

Published 12 November 2021, *Sci. Adv.* **7**, eabi6439 (2021)
DOI: 10.1126/sciadv.abi6439

This PDF file includes:

Table S1
Figs. S1 to S5
